# Supplementary material for: Research on robust adaptive control of strong nonlinear complex large power grids
Source: PLoS One. 2024 Jun 14;19(6):e0300099. doi: 10.1371/journal.pone.0300099 (PMC11178179; doi:10.1371/journal.pone.0300099)
Supplement: Attachment — Submitted filename: Comments_PONE-D-23-43214.pdf [file pone.0300099.s001.pdf]

Comment

In this work, a Multi-Index Nonlinear Robust Adaptive Control (MINRAC) method has been proposed for ultra-complex multi-input multi-output power systems with uncertain parameter factors and external disturbances. However, in order to further improve the quality of this manuscript, the following comments should be carefully considered:

1. The manuscript has some typos, ambiguous sentences and language issue that require full spell and syntax checking. The content in the abstract should be further condensed to highlight the innovative points of this manuscript.
2. In introduction, the research background should be further strengthened and the descriptions of existing research should be further summarized. In order to make it easier for readers to grasp the key points, the contributions should be refined and listed point by point.
3. There has been relatively little literature research in recent years.
4. Moreover, some expressions should be added with corresponding references, as they are not the first time proposed, such as:

$$\begin{cases} \dot{\delta}_i = \omega_0(\omega_i - 1) \\ \dot{\omega}_i = \dfrac{1}{T_{ji}}(P_{mi} - P_{ei}) - \dfrac{D_i}{T_{ji}}(\omega_i - 1)(i = 1,2,\cdots m) \\ \dot{E}'qi = -\dfrac{1}{T'_{doi}}E_{\mathrm{qi}} + \dfrac{K_i}{T'_{\mathrm{doi}}}U_i + d_i \end{cases} \quad (1)$$

5. The text size in formula 18 is inconsistent, please carefully check the whole manuscript. And the information about the coordinate axes in some figures is missing, please carefully check them.
6. In the validation section, other algorithms are compared. Authors should elaborate on the fairness and reasonableness of comparison in detail.
7. Some existing methods, which are widely utilized these days to solve several real world problems including the described issues, multilayer neurocontrol of high-order uncertain nonlinear systems with active disturbance rejection, asymptotic tracking with novel integral robust schemes for mismatched uncertain nonlinear systems and so on.
8. The conclusions should be further condensed.
