# Supplementary material for: Research on robust adaptive control of strong nonlinear complex large power grids
Source: PLoS One. 2024 Jun 14;19(6):e0300099. doi: 10.1371/journal.pone.0300099 (PMC11178179; doi:10.1371/journal.pone.0300099)
Supplement: Attachment — Submitted filename: Response to Reviewers.docx [file pone.0300099.s002.docx]

**Comments to the authors of academic editor:**

*The reviewers recommend reconsideration the manuscript with revision and modification.I invite the authors to resubmit the manuscript after addressing the comments raised by the reviewers.*

**Reply:** Many thanks for your kind handling of the review of our paper. Following your suggestions,we have carefully revised the paper by clarifying the given questions as explained below.

**Comments to the authors of Reviewer #1:**

*1. What is an* *"ultra-complex multiple-input multiple-output power system"? Please explain in detail in the text.* **Reply:** "Ultra-complex multiple-input multiple-output power system" mainly refers to the smart grid system that can connect the major coal power base, the major hydropower base, the major nuclear power base and the major renewable energy base.And the content had been added in “1 INTRODUCTION” on Page 1.

*2. To make it easier for the reader to understand, please explain the variables that appear for the first time in the paper. In addition, there are some errors in the symbols* *in the paper. It is recommended to check the symbols throughout the paper.* **Reply:** We have added the variables that appear for the first time in the paper in “NOMENCLATURE” on Page 1,and corredted the errors of symbols in the paper.

*3. What does the MINRC in section IV IEEE 3-MACHINE 9-NODE SYSTEM stand for? To avoid confusing the reader, please explain it clearly in the paper.*

**Reply:** IEEE 3-machine 9-node system is a classic power system model, consisting of 9 nodes and 12 branches.The system is characterized by its moderate size and the inclusion of common power system components, so it is often used in academic research and engineering practice.And it was added on Page 7.

*4. The authors claim that the proposed control method can ensure that the multiple indexes concerned by ultra-complex power systems can be controlled at their expected values under the condition of the uncertainty of system parameters. However, the authors said in the simulation that the system parameters are accurate. This is confusing operation, please make a detailed explanation.*

**Reply:** In order to verify the effectiveness of MINRAC control law,the control law was applied to IEEE 3-machine 9-nodesystem.The data of line,generator and load were shown in Table 1~Table 4,and the unit of parameters in the table was the standard unit value.Fig.5(a)-(d) showed that both MINRC and MINRAC could maintain the terminal voltage and output active power of the generator at the original given value.Due to the change of network parameters,the power angle of the motor was bound to change to adapt to the new equilibrium point when the terminal voltage and active power remained unchanged.

Then we can make sure that inn multi-machine power system,for the uncertainty of the generator itself,such as damping coefficient,rotor inertia time constant is inaccurate and generator excitation of electromagnetic disturbance,MINRAC method effect rather than in a single machine infinite system,the mechanism of selection by the output function system of the relationship between indicators such as angle,angular frequency and voltage of the machine as a constraint.By adjusting the uncertain parameter value by adaptive control,these indexes are forced to tend to the given expected value,so as to achieve static tracking.

5. *The authors should* *add some more recent relevant references in the references section and cite them properly in the paper.* **Reply:** We have added and renewed 4 relevant references in the references section and cite them properly in the paper.And the content had been added in “1 INTRODUCTION” on Page 2.

*6. It is recommended that the author reformulate an appropriate title based on the content of the paper.*

**Reply:** Many thanks for your encouraging comments.In this paper,a Multi-Index Nonlinear Robust Adaptive Control (MINRAC) method was proposed for ultra-complex multi-input multi-output power systems with uncertain parameter factors and external disturbances,and the controller designed by this method has excellent static and dynamic characteristics.After our unanimous discussion,we think that the research content conforms to the content of the article,so we do not change the title of “**Research on Robust Adaptive Control of Strong Nonlinear Complex Large Power Grids**” . *7.* *In conclusion part, more* *future works and challenges should be* *recommended.* **Reply:** Future works and challenges have been recommended in conclusion part on Page 9.

*8. To improve the quality of the paper, it is recommended to improve the English writing skills.*

**Reply:** According to your suggestion,we have correct grammatical mistakes of the manuscript.

**Comments to the authors** **of reviewer #2:**

*In this work, a Multi-Index Nonlinear Robust Adaptive Control (MINRAC) method has been proposed for ultra-complex multi-input multi-output power systems with uncertain parameter factors and external disturbances. However, in order to further improve the quality of this manuscript, the comments in the uploaded file should be carefully considered.*
 **Reply:** Many thanks for your encouraging comments.We have considered the comments in the uploaded files,and we also have carefully revised the paper.
